# p22phox confers resistance to cisplatin, by blocking its entry into the nucleus

**DOI:** 10.18632/oncotarget.2893

**Published:** 2015-02-19

**Authors:** Chih-Chang Hung, Chen-Yu Chien, Wei-Fan Chiang, Chang-Shen Lin, Tzyh-Chyuan Hour, Hau-Ren Chen, Ling-Feng Wang, Jenq-Yuh Ko, Chi-Hua Chang, Jeff Yi-Fu Chen

**Affiliations:** ^1^ Graduate Institute of Medicine, College of Medicine, Kaohsiung Medical University, Kaohsiung, Taiwan; ^2^ Department of Biotechnology, Kaohsiung Medical University, Kaohsiung, Taiwan; ^3^ Department of Otorhinolaryngology, School of Medicine, College of Medicine, Kaohsiung Medical University, Kaohsiung, Taiwan; ^4^ Department of Otorhinolaryngology, Kaohsiung Medical University Hospital, Kaohsiung, Taiwan; ^5^ Department of Otorhinolaryngology, Kaohsiung Municipal Hsiao-Kang Hospital, Kaohsiung Medical University, Kaohsiung, Taiwan; ^6^ Department of Dentistry, Chi-Mei Medical Center, Liouying, Taiwan; ^7^ Department of Dentistry, School of Dentistry, National Yang-Ming University, Taipei, Taiwan; ^8^ Department of Biochemistry, Kaohsiung Medical University, Kaohsiung, Taiwan; ^9^ Department of Life Science and Institute of Molecular Biology, National Chung Cheng University, Chia-Yi, Taiwan; ^10^ Department of Otorhinolaryngology, Kaohsiung Municipal Ta-Tung Hospital, Kaohsiung Medical University, Kaohsiung, Taiwan; ^11^ Department of Otolaryngology, National Taiwan University, College of Medicine, Taipei, Taiwan; ^12^ Department of Dentistry, Chang Gung Memorial Hospital, Kaohsiung, Taiwan

**Keywords:** p22phox, CDDP resistance, apoptosis, PI3K/Akt, oral squamous cell carcinoma (OSCC)

## Abstract

Cisplatin (CDDP) is a potent chemotherapeutic agent but resistance to the drug remains a major challenge in cancer treatment. To evaluate the efficacy of CDDP in oral squamous cell carcinoma (OSCC), we found that p22phox was highly expressed in CDDP-resistant OSCC specimens. Knockdown of p22phox sensitized OSCC cell lines to CDDP (*P* < 0.05). Stable overexpression of p22phox augmented CDDP resistance, as evidenced by the significantly higher IC_50_ values. This cytoprotective effect was attributed to the abrogation of CDDP-induced apoptosis. Akt phosphorylation was increased in p22phox stable lines. However, blocking PI3K/Akt pathway only partially restored CDDP-induced apoptosis. In addition, the overexpressed p22phox in OSCC cells exhibited cytoplasmic localization with enhanced perinuclear expression, consistent with the localization pattern in OSCC specimens. Remarkably, CDDP entry into the nucleus was severely impaired in p22phox-overexpressing cells (*P* < 0.001), and cytoplasmically accumulated CDDP was co-localized with overexpressed p22phox. This was supported by decreased CDDP-DNA adduct formation and delayed chk1-p53 signaling activation. Together, overexpression of p22phox sequestered CDDP and caused defective CDDP entry into the nucleus, significantly attenuating CDDP-induced apoptosis. Such diminished apoptosis was further abolished by p22phox-activating PI3K/Akt pathway. Our work has suggested a novel biomarker and insight into the mechanism of CDDP resistance.

## INTRODUCTION

NADPH oxidases are a major source of reactive oxygen species (ROS) production in phagocytic leukocytes and in many non-phagocytic cells [1]. The phagocytic NADPH oxidase mediates oxidative stress, contributing to the killing of the invading microorganisms. In non-phagocytic cells, in addition to producing ROS, NADPH oxidases can regulate diverse physiological processes including cell proliferation, differentiation and death. The phagocytic and non-phagocytic homologs constitute the NOX family NADPH oxidases with a total of seven members in human: NOX1, NOX2 (phagocytic NADPH oxidase), NOX3, NOX4, NOX5, DUOX1 and DUOX2. Activation of most NOX enzymes and generation of ROS require the assembly with numerous regulatory proteins, functioning as multicomponent enzymatic complexes. p22phox is one of the regulatory proteins whose major function is to stabilize the NOX enzymes to which it binds. This is supported by the studies showing that p22phox down-regulation results in decreased activity of several NOX enzymes [2, 3]. Despite being a key modulator for NOX enzymatic activity, the role of p22phox in cancer progression is relatively unknown. In human renal cell carcinoma, p22phox may promote carcinogenesis by inactivating a tumor suppressor protein [4]. Up-regulation of p22phox represents a pro-survival, anti-apoptotic signal in pancreatic cancer cells [5]. Furthermore, there is evidence that NADPH oxidases are the major sources of ROS in oral squamous cell carcinoma (OSCC) [6]. It is unknown whether p22phox has an impact on the treatment of OSCC.

Even though long-term exposure inevitably develops chemoresistance, cis-diamminedichloroplatinum (II) (CDDP, cisplatin) is still widely used for the treatment of various solid tumors, including testicular, ovarian, breast, lung, bladder and cervical cancers [7]. To date, CDDP-based chemotherapy combined with other chemotherapeutic agents remains the first-line treatment for oral cancer patients [8, 9]. For example, CDDP combined with 5-fluorouracil (5-FU) potentiates the induction of apoptosis in oral cancer cells [10] and gives improved survival of patients with advanced OSCC [11]. In addition, EGFR overexpression is involved in CDDP resistance in esophageal adenocarcinoma [12], and combined treatments with CDDP and EGFR inhibitors show enhanced susceptibility to CDDP-mediated apoptosis in OSCC cells [13]. On the other hand, accumulated level of ROS has been shown to increase CDDP-induced cancer cell cytotoxicity. Interestingly, there is a report indicating that CDDP induces ROS via activation of NADPH oxidases in prostate cancer cells [14]. However, whether p22phox, the major modulator for the activation of NADPH oxidases, might play a role in CDDP resistance of oral cancer has to be elucidated.

In this study, up-regulation of p22phox was observed in CDDP-resistant but not CDDP-sensitive OSCC tissue samples. We then hypothesized that p22phox was a CDDP-resistant gene in oral cancer. To test this hypothesis, we used siRNA-mediated gene silencing to evaluate the effect of p22phox expression in CDDP sensitivity. Two independent p22phox stable lines were established and their IC_50_ values for CDDP treatment were determined. Later, the detailed mechanism underlying p22phox-dependent CDDP resistance in OSCC cells was thoroughly investigated. Overall, our results suggest that p22phox is a CDDP-resistant gene that suppresses DNA adduct-induced apoptosis by blocking CDDP uptake into the nucleus and activating PI3K/Akt pathway.

## RESULTS

### Up-regulation of p22phox expression in CDDP-resistant OSCC tissues

**Figure 1:**
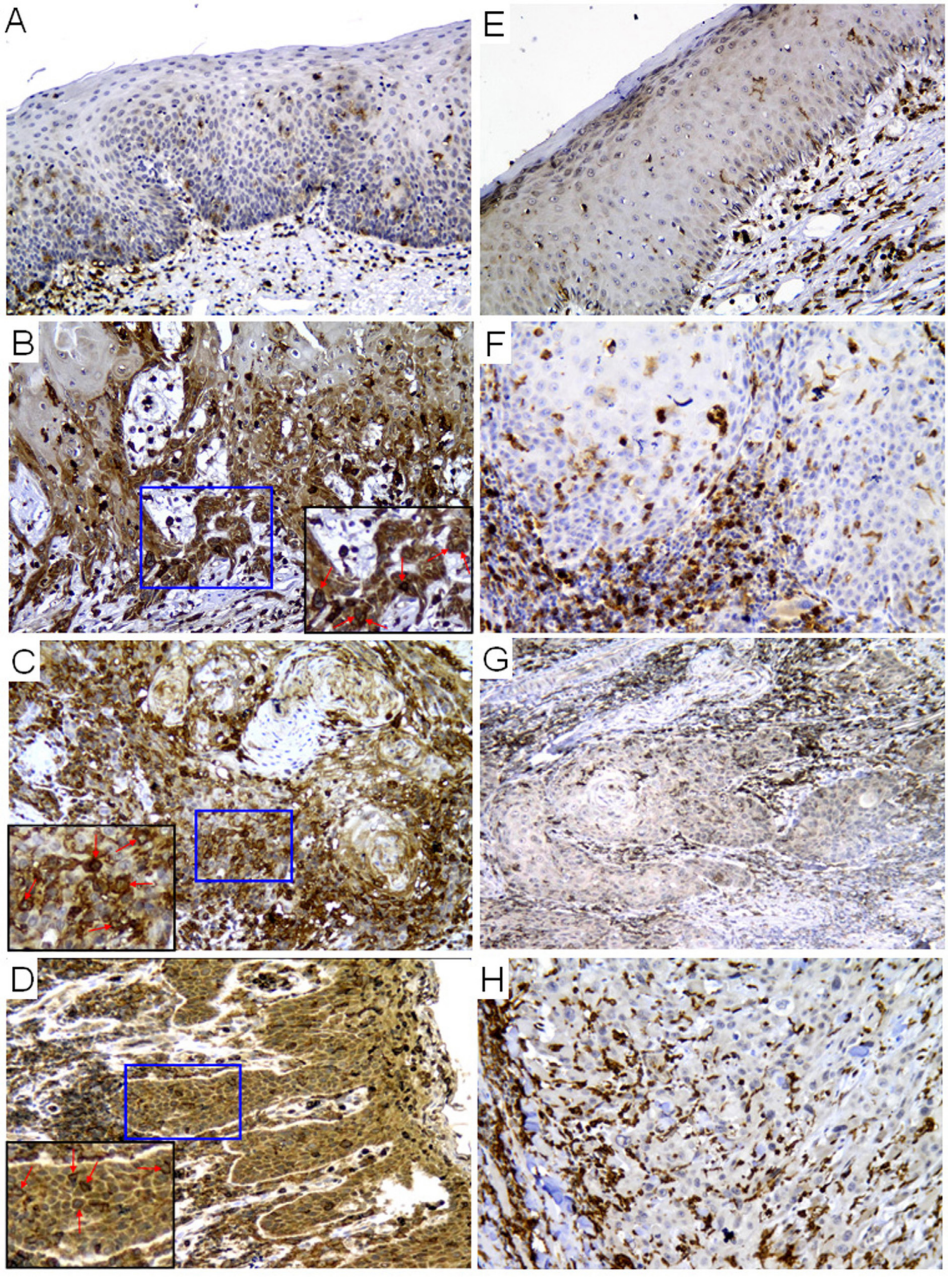
Expression of p22phox was significantly higher in CDDP-resistant than CDDP-sensitive OSCC tissues Immunohistochemical staining of p22phox protein expression in healthy mucosa **(A and E)**, CDDP-resistant **(B–D)** and CDDP-sensitive OSCC **(F–H)** tissue sections. The magnified views of the boxed (blue) areas are shown in the corner panels (black). The red arrows indicate intense perinuclear staining of p22phox in the CDDP-resistant OSCC cases. Magnification: 200X.

To understand the correlation between p22phox and clinical efficacy of CDDP, we used immunohistochemistry (IHC) to detect p22phox expression in three cases of CDDP-resistant and CDDP-sensitive OSCC, respectively. There was negative expression of p22phox in two cases of healthy mucosa (Figure 1A and 1E). Moreover, we found that p22phox was highly expressed in carcinoma areas of the three CDDP-resistant cases (Figure 1B–1D or Table 1, patients 4–6), but was nearly absent in those of the three CDDP-sensitive cases (Figure 1F–1H or Table 1, patients 1–3). Interestingly, the CDDP-resistant cases displayed cytoplasmic localization of p22phox with enhanced staining surrounding the nucleus (indicated by arrows in Figure 1B–1D). It is also notable that, in all OSCC cases, p22phox was abundantly expressed in phagocytic leukocytes that either resided in the stroma or infiltrated into the carcinoma areas. Since phagocytic leukocytes are the canonical expression sites of p22phox, this helped to validate the p22phox staining in carcinoma cells. These results suggested that up-regulation of p22phox might confer resistance to CDDP in OSCC patients.

**Table 1:** Clinicopathological information of the CDDP-sensitive and CDDP-resistant OSCC patients

Patient	Age	Site	Stage	Primary date	Primary therapy*	Recurrence	Expiry
1	50	Retromolar trigone	IV	2009/05/30	S+CCRT		
2	55	Oral tongue	IV	2010/10/24	S+CCRT		
3	59	Buccal mucosa	IV	2011/05/04	S+CCRT		
4	56	Buccal mucosa	IV	2010/05/31	S+CCRT	2010/08/09	2010/10/31
5	49	Buccal mucosa	IV	2010/07/27	S+CCRT	2010/11/24	2011/06/07
6	48	Buccal mucosa	III	2009/10/06	S+C/T	2009/12/29	2010/02/11

* S, surgical excision; CCRT, concurrent chemoradiotherapy; C/T, chemotherapy

### Knockdown of p22phox enhances CDDP cytotoxicity in OSCC cell lines

**Figure 2:**
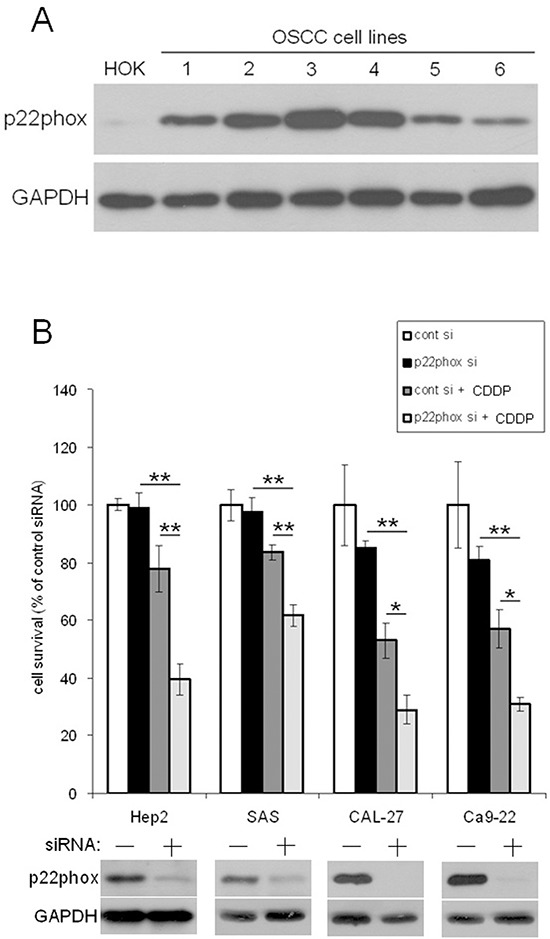
Down-regulation of p22phox increased sensitivity to CDDP-induced cytotoxicity **(A)** The endogenous expression of p22phox (22 kDa) in six oral cancer cell lines and HOK was analyzed by immunoblotting. The six OSCC cell lines: 1, SAS; 2, Hep2; 3, Ca9–22; 4, CAL-27; 5, HSC-3; 6, FaDu. **(B)** The expression of p22phox in Hep2, SAS, CAL-27 and Ca9–22 was knocked down by siRNA, and the cells were evaluated for survival under CDDP treatment. The cells were transfected with p22phox siRNA oligos (50 nM) for 48 h, followed by treatment with the various concentrations of CDDP (5 μM for Hep2 and SAS, 7.5 μM for CAL-27 and 10 μM for Ca9–22) for another 24 h. Knockdown of p22phox in the four cell lines was confirmed by Western blotting shown in the bottom panels. Survived cells were calculated by trypan blue staining. The viability of cells transfected with scrambled siRNA (control siRNA) was deliberately set to 100%. Each value is the average ± SD of three independent measurements. The experiment was repeated at least three times and the representative data are shown. *P < 0.05 and **P < 0.001 indicated significant difference from the co-treatment of CDDP and p22phox siRNA. Abbreviations: cont, control; si, siRNA.

To examine the role of p22phox in CDDP resistance, we showed that p22phox was abundant in six OSCC cell lines but was only moderately expressed in human oral keratinocyte (HOK) (Figure 2A), consistent with the IHC results (Figure 1A and 1E). Knockdown of p22phox expression by siRNA in four OSCC cell lines with relatively higher p22phox levels sensitized the cells to CDDP treatment and further significantly decreased cell survival (Figure 2B) (*P* < 0.001 in Hep2 and SAS; *P* < 0.05 in CAL-27 and Ca9–22). These data indicated that p22phox was selectively expressed and required for CDDP resistance in OSCC cells.

### Overexpression of p22phox confers cytoprotection against CDDP in OSCC cells

**Figure 3:**
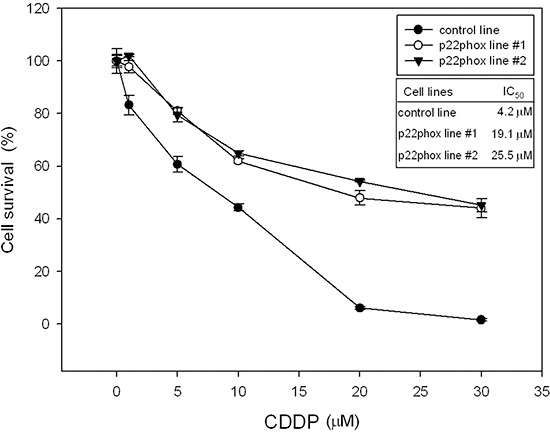
Increased survival rates of p22phox stable lines treated with CDDP Oral cancer cells stably expressing p22phox (p22phox line #1 and #2) were treated with increasing concentrations (1, 5, 10, 20 and 30 μM) of CDDP for 48 h. Survived cells were analyzed by MTT assay. The control line was transfected with an empty expression vector and its viability was deliberately set to 100%. The IC_50_ values for CDDP were determined by CalcuSyn 2.1 software. All measurements were performed in triplicate and expressed as mean ± SD. The experiment was repeated at least three times and the representative data are shown.

We next confirmed the effect of p22phox in CDDP efficacy by establishment of stable p22phox expression in OSCC cells. SAS cells were transfected with a p22phox-red fluorescent tag (DsRed) expression construct and selected by G418 (2 mg/ml) for stable transfectants. After one month, two independent stable clones (p22phox line #1 and #2) were obtained. Both stable cell lines had higher survival rates than the control line (DsRed only) under increasing concentrations of CDDP treatment. Moreover, the IC_50_ values for CDDP were at least 4.5-fold higher in p22phox stable lines than that in the control line; 19.1 or 25.5 μM vs. 4.2 μM (Figure 3). Thus, overexpression of p22phox could confer cytoprotective effect and rescue cell survival against CDDP-induced cell death in OSCC cells.

### p22phox stable lines are protected against CDDP-induced apoptosis

**Figure 4:**
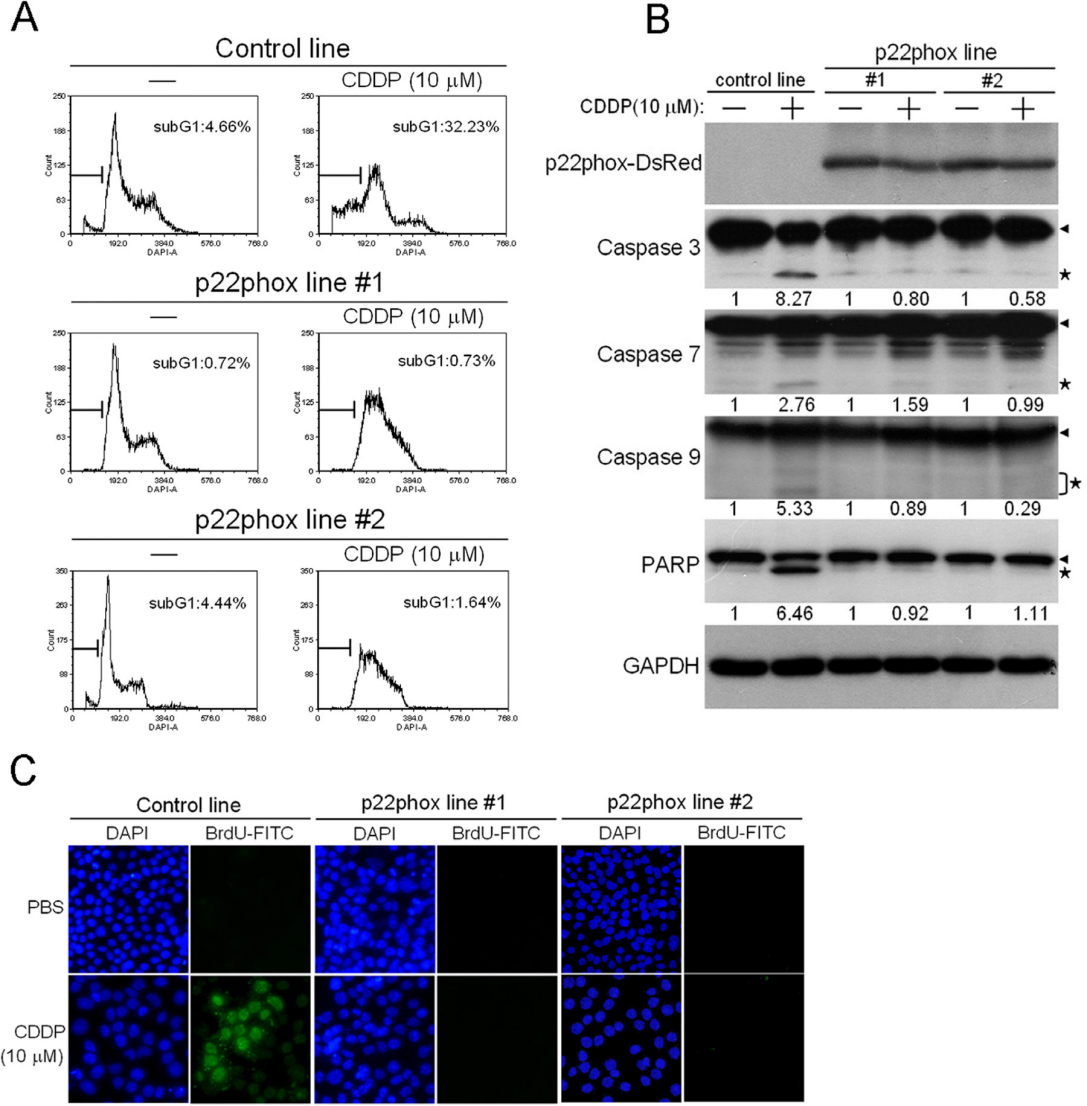
Abolishment of CDDP-induced apoptosis in p22phox stable lines **(A)** Cells were treated with 10 μM CDDP for 24 h, and then subG1 population was evaluated by flow cytometry. The proportion of subG1 cells was presented as the percentage of the entire cell population. **(B)** Cells were treated with CDDP (10 μM) for 48 h and the expression of p22phox-DsRed fusion protein (50 kDa), caspase 3 (35 kDa), caspase 7 (35 kDa), caspase 9 (47 kDa) and PARP (116 kDa) was revealed by Western blot analysis. p22phox-DsRed fusion protein was detected using anti-DsRed antibody. Arrow heads and stars indicate the pro-forms and the cleaved forms of the caspase proteins and PARP, respectively. The numbers below the blots were quantitative ratios of cleaved caspase 3 (17 kDa), caspase 7 (20 kDa), caspase 9 (35/37 kDa) or PARP (89 kDa) /GAPDH band densities under CDDP treatment normalized to those without CDDP treatment. **(C)** DNA fragmentation of the CDDP-treated cells was detected by the TUNEL assay using fluorescence microscopy. Magnification: 200X. All experiments were repeated four times, and the representative data are shown.

Because CDDP is known to induce apoptosis in cancer cells [15, 16], we investigated whether such effect was attenuated in p22phox-overexpressing cells. Cell cycle analysis by DAPI staining showed that CDDP treatment in the control line caused a significant increase in subG1 cell population (4.66% to 32.23%), indicating the induction of apoptosis by CDDP. In sharp contrast, CDDP-induced subG1 accumulation was nearly abolished in p22phox stable lines (Figure 4A). We then examined whether this protective effect involved attenuation of apoptotic signaling. Whereas the cleaved forms of caspase 3, caspase 7 and caspase 9 were induced by CDDP in the control line, such induction was virtually abrogated in the two p22phox stable lines. The cleaved form of PARP, a well-known substrate for caspase 3 cleavage during apoptosis, was dramatically induced by CDDP in the control line. However, CDDP had no effect on the induction of PARP cleavage in the stable lines (Figure 4B). Furthermore, we showed that the majority of cells in CDDP-treated control line were TUNEL-positive, while no detectable TUNEL staining was observed in CDDP-treated p22phox stable lines (Figure 4C). Together, these results strongly suggested that overexpression of p22phox could protect OSCC cells from CDDP-induced apoptosis.

### p22phox counteracts CDDP-induced apoptosis through PI3K/Akt pathway

**Figure 5:**
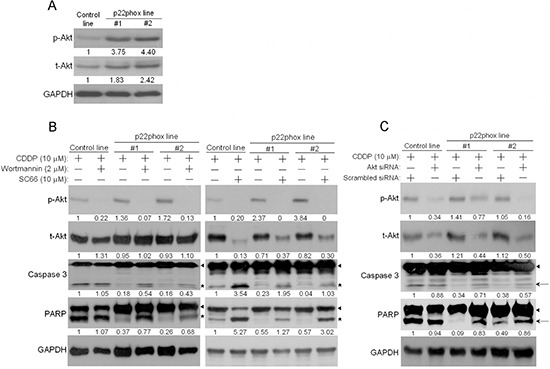
Elevated PI3K/Akt activity contributed to inhibition of CDDP-induced apoptosis in p22phox stable lines **(A)** The endogenous expression level of phosphorylated Akt (p-Akt) in p22phox stable lines was revealed by Western blot analysis. The numbers below the blots were quantitative ratios of p-Akt (56 kDa) or total Akt (t-Akt, 56 kDa)/GAPDH band intensities in p22phox lines normalized to those of control line. **(B)** PI3K/Akt activity was blocked by wortmannin or SC66, and p-Akt, t-Akt and cleavage of caspase 3 and PARP were examined. Cells were pretreated with wortmannin (2 μM) overnight or SC66 (10 μM) for 1 h, followed by the combined treatments of CDDP (10 μM) and the inhibitors for another 24 h. Arrow heads and stars represent the pro-forms and the cleaved forms of caspase 3 and PARP, respectively. The numbers below the blots were quantitative ratios of p-Akt, t-Akt, cleaved caspase 3 or cleaved PARP/GAPDH band intensities normalized to those without the inhibitor treatments in control line. **(C)** The cells were transfected with Akt or scrambled siRNA oligos (100 nM) for 48 h, followed by treatment with CDDP for 24 h. The cell lysates were analyzed by Western blotting and the results were similarly quantified as in (B). Arrow heads and arrows indicate the pro-forms and the cleaved forms of caspase 3 and PARP, respectively. The experiments were repeated four times, and the representative data are shown.

We then asked how p22phox inhibited CDDP-induced apoptosis in OSCC cells. PI3K/Akt pathway was examined because it is known to transduce pro-survival and anti-apoptotic signals in cells. There were much higher levels of endogenous Akt phosphorylation (p-Akt; S473) in p22phox stable lines, implicating increased Akt activity (Figure 5A). To determine whether this high Akt activation contributed to CDDP resistance, we tested how p22phox stable lines would respond to CDDP in the presence of the PI3K/Akt inhibitor wortmannin, SC66 or 3-MA (Supplementary Figure 1). In both stable lines, CDDP-induced apoptosis was significantly rescued when Akt activity was inhibited, as evidenced by the marked decrease in p-Akt and the induction of cleaved caspase 3 and PARP (Figure 5B and Supplementary Figure 1). Noticeably, total Akt (t-Akt) levels in SC66-treated cells were also reduced presumably because of ubiquitination-mediated protein degradation when Akt was inactivated by the inhibitor [17]. Furthermore, consistent with the results in Figure 5B, the levels of cleaved caspase 3 and PARP were increased in response to CDDP treatment when Akt expression was knocked down by siRNA in p22phox stable lines (Figure 5C). Therefore, inhibition of CDDP-induced apoptosis in p22phox stable lines was, at least in part, mediated through PI3K/Akt signaling pathway.

### Subcellular protein localization of the ectopically overexpressed p22phox in OSCC cells

**Figure 6:**
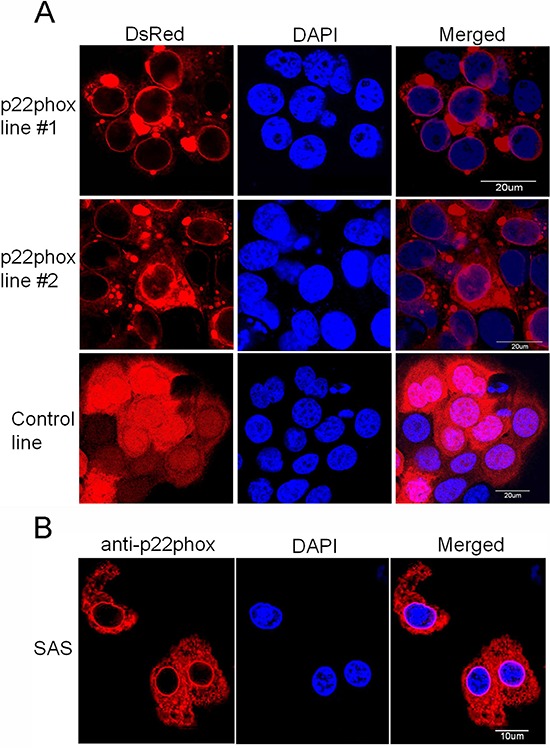
Overexpression of p22phox led to its perinuclear localization in OSCC cells **(A)** The fluorescence signal of p22phox-DsRed in the stable lines or DsRed only in the control line was visualized by confocal microscopy. p22phox-DsRed fusion protein was mostly localized to the perinuclear region, yielding the ring-like pattern. In contrast, DsRed protein was localized throughout the whole cell in a diffuse fashion. **(B)** Overexpression of p22phox in the parental SAS cells by introducing a non-tagged p22phox expression construct (pcDNA3.0-p22phox) resulted in a similar perinuclear localization revealed by immunofluorescence microscopy. Magnification: 1000X. The experiments were repeated four times, and the representative images are shown.

The expression site of the ectopic p22phox protein in p22phox stable lines was examined. Since p22phox was tagged with the red fluorescence protein DsRed, we directly visualized the subcellular localization of p22phox by fluorescence microscopy in living cells. While there was diffuse DsRed fluorescence signal throughout the entire cell in the control line, the overexpressed p22phox-DsRed was predominantly localized in the cytoplasm, forming a ring-like pattern at the nuclear periphery in p22phox stable lines (Figure 6A). To confirm this observation, the parental SAS cells were transiently transfected with a non-tagged p22phox expression construct (pcDNA3.0-p22phox) and analyzed by immunofluorescence microscopy. Consistently, p22phox expression detected by anti-p22phox antibody displayed similar staining pattern around the nucleus (Figure 6B). More importantly, these findings are reminiscent of those in clinical samples in which p22phox was intensively localized to the perinuclear area when overexpressed in the CDDP-resistant specimens (Figure 1B–1D).

### Blockade of CDDP uptake into the nucleus leads to decreased CDDP-DNA adduct formation and attenuated chk1-p53 activation in p22phox-overexpressing cells

**Figure 7:**
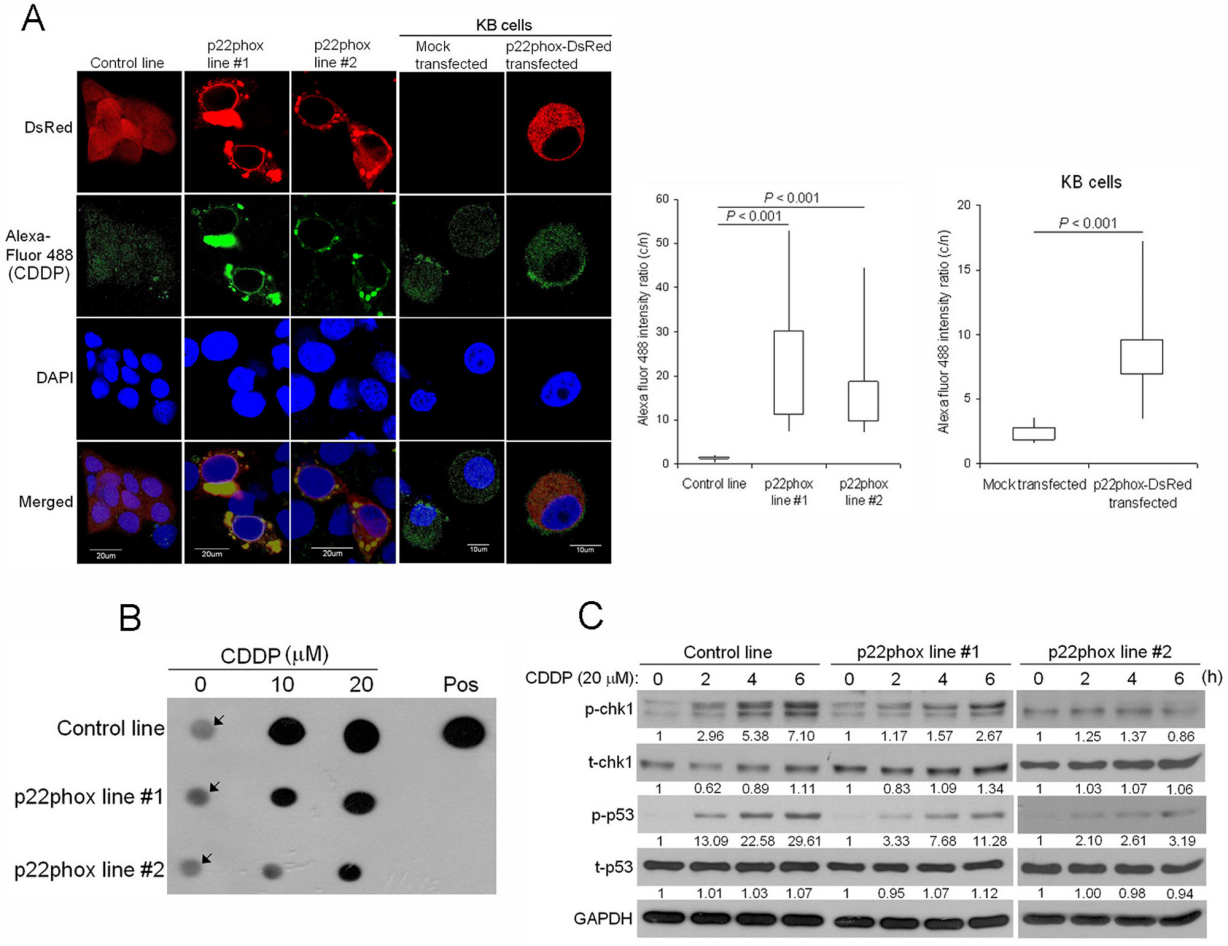
Reduced CDDP uptake into the nucleus, CDDP-DNA adduct formation and chk1-p53 activation in p22phox stable lines **(A)** CDDP distribution and localization was monitored by Alexa Fluor 488 fluorescence in p22phox stable lines and p22phox-overexpressing KB cells using confocal microscopy. Magnification: 1000X. The respective cytoplasmic and nuclear fluorescence intensity in each cell was measured by Olympus Fluoview Viewer (Ver. 3.0 software). The average cytoplasm-to-nucleus intensity ratios were determined from the randomly selected 24, 20, 21, 21 and 12 cells in control line, p22phox stable line #1, p22phox stable line #2, mock transfected KB and p22phox-DsRed transfected KB, respectively. The quantitative results and statistical analysis were shown in the right panels. **(B)** The p22phox stable lines and the control line were treated with 10 or 20 μM CDDP overnight, and genomic DNA was isolated and analyzed by dot blot assay using anti-CDDP adducts antibody. The arrows denote non-specific background signals. **(C)** The cells were treated with CDDP (20 μM) for 0, 2, 4 or 6 h and the lysates were analyzed by Western blot analysis using antibodies against t-chk1, p-chk1, t-p53 and p-p53. The numbers below the blots were quantitative ratios of p-chk1, t-chk1, p-p53 or t-p53/GAPDH band intensities normalized to those without CDDP treatment. Abbreviations: c, cytoplasm; n, nucleus; pos, positive control. The experiments were repeated four times, and the representative images or data are shown.

The unique localization pattern of p22phox when overexpressed propelled us to investigate whether this could contribute to p22phox-dependent CDDP resistance. Using green fluorescence (Alexa Fluor 488)-labeled CDDP to monitor CDDP uptake and distribution, we found that Alexa Fluor 488-CDDP signal was apparently more intense in the cytoplasm than the nucleus in p22phox stable lines. In contrast, the fluorescence signal was uniformly distributed throughout the entire cell in the control line. Furthermore, these observations were reproduced in KB carcinoma cell line transiently overexpressing p22phox (p22phox-DsRed). Quantitative analysis revealed that the average cytoplasm-to-nucleus ratio of Alexa Fluor 488 intensity was significantly higher in the p22phox-overexpressing cells; 22.31 and 16.58 vs 1.22, *P* < 0.001 in the stable lines and 8.47 vs 2.41, *P* < 0.001 in KB cells. More remarkably, the accumulated Alexa Fluor 488-CDDP in the cytoplasm coincided with the localization sites of the ectopically expressed p22phox, including those of the ring-like structure, in both cell lines (Figure 7A). To evaluate whether accumulation of CDDP in the cytoplasm would lead to decreased DNA damage in the nucleus, we examined CDDP-DNA adducts in p22phox stable lines. Indeed, dot blot analysis using anti-CDDP adduct antibody showed significant reduction of DNA adduct formation compared to the control line when the cells were treated with increasing doses of CDDP (Figure 7B). Furthermore, the activation of checkpoint kinase chk1 and p53, the two indicators of DNA damage response, was either attenuated or delayed during the 6-h CDDP treatment period in p22phox stable lines, as indicated by the decreased induction of p-chk1 (Ser345) and p-p53 (Ser15) (Figure 7C). These findings suggested that overexpression of p22phox might block the entry of CDDP into the nucleus, resulting in reduced formation of DNA adducts and activation of DNA repair signaling.

## DISCUSSION

Although the NOX family NADPH oxidases and the key modulator p22phox have been implicated in cancer development [4–6, 18], it is still unknown about their role in cancer drug resistance. Since NADPH oxidases are the major source of ROS production, we previously used p22phox as the marker to investigate the correlation between ROS and oral carcinogenesis. Whereas there was no significant correlation between p22phox expression and oral cancer progression, we surprisingly found that p22phox appeared to be preferentially up-regulated in CDDP-resistant OSCC specimens. On the contrary, there was little p22phox expression in three CDDP-sensitive OSCC tissues and in healthy mucosa. These results motivated us to hypothesize that p22phox was involved in CDDP resistance of OSCC. We further tested the hypothesis by *in vitro* studies. While p22phox was abundantly expressed in all six OSCC cell lines, it was almost absent in normal oral keratinocytes (HOK). Knockdown of p22phox expression had little effect on OSCC cell survival, but significantly sensitized the cells to CDDP-induced cytotoxicity. Interestingly, we noticed that the combined cytotoxic effect of p22phox knockdown and CDDP treatment was greater than the sum of their individual effect; for example, in Hep2 cells, the combined treatment contributed to 60% cytotoxicity while the sum of the separate treatments accounted for only 23% cytotoxicity. It is thus plausible that there was a synergistic, more than additive, cytotoxic effect resulted from concurrent challenge with p22phox down-regulation and CDDP treatment in oral cancer cells.

If knockdown of p22phox sensitized OSCC cells to CDDP, we reasonably predicted that overexpression of p22phox should reverse such effect. Indeed, after establishment of stable p22phox expression in SAS OSCC cells, we found that the two stable lines became much more resistant to increasing concentrations of CDDP treatment. Consistent with these results, the IC_50_ values of CDDP in p22phox stable lines were at least 4.5-fold higher than that in the control line. To further support these data, we generated additional p22phox-expressing stable clones whose parental line was Hep2, and then evaluated their response to CDDP. Cell survival was significantly rescued under CDDP treatment (10 μM) for 24 h in the stable clones (*P* < 0.01) (Supplementary Figure 2). These results indicate that overexpression of p22phox can enhance resistance to CDDP, thus protecting OSCC cells from CDDP-mediated killing. To understand the mechanism behind the protective effect against CDDP-induced cytotoxicity, we examined whether cell cycle progression was perturbed in CDDP-treated p22phox stable lines. The results suggest that p22phox overexpression might fully prevent OSCC cells from CDDP-induced apoptotic death, thus conferring the cytoprotective effect. We noticed that, especially in p22phox stable lines, CDDP appeared to induce a severe blockade of S-phase progression in cell cycle (Figure 4A, middle and lower right panels). This is consistent with previous reports that CDDP could induce S-phase arrest as a consequence of S-phase checkpoint activation in many cancer cell types [19, 20]. Interestingly, another previous study showed that increased CDDP resistance might be associated with augmented CDDP-induced S-phase arrest [21]. However, it is unclear how p22phox overexpression aggravated S-phase progression in CDDP-treated OSCC cells.

It has been long known that PI3K/Akt signaling pathway mediates suppression of apoptosis, thereby promoting cell survival [22, 23]. Indeed, basal Akt activity was higher in p22phox stable lines, prompting us to test whether it was this increased Akt activity that counteracted CDDP-induced apoptotic signaling. By using PI3K/Akt selective inhibitors and Akt specific siRNA, we showed significant restoration of the apoptotic signaling in both stable lines, suggesting the involvement of PI3K/Akt pathway in this biological effect. Noticeably, Akt allosteric inhibitor SC66 enhanced CDDP-induced apoptosis in the control line, indicating that basal Akt activity could contribute to intrinsic CDDP resistance in OSCC cells. Together, these results are consistent with recent studies that Akt played an important role in promoting CDDP resistance in various cancers [24–27]. Still, it has not been demonstrated how Akt activity might be regulated by p22phox in OSCC cells. Several lines of evidence suggest that ROS can promote Akt activation, leading to induction of carcinogenic effects [28–30]. Furthermore, there is a report that up-regulation of p22phox resulted in increased Akt activity likely through a ROS-mediated mechanism in renal carcinoma cells [4]. However, despite being the major modulator for NADPH oxidases activation, p22phox overexpression alone had little effect on ROS level in OSCC cells (unpublished results). More intriguingly, we also showed that the ectopically overexpressed p22phox in OSCC cells was largely co-localized with endoplasmic reticulum (ER) (Supplementary Figure 3). Furthermore, previous studies reported that ER stress could significantly induce Akt activation, contributing to CDDP and doxorubicin resistance in liver and lung cancer cells, respectively [31, 32]. These results have raised the possibility that p22phox can activate Akt through an ER stress-dependent pathway in OSCC cells. Detailed mechanisms are currently under investigation.

In Figure 5B, even complete inhibition of Akt phosphorylation (or activity) by SC66 (right panel) in p22phox stable lines was unable to yield similar level of cleaved caspase 3 as in the control line. These data clearly implied that there was additional mechanism (s) involved in p22phox-dependent CDDP resistance in OSCC cells. The unique perinuclear p22phox staining pattern in CDDP-resistant OSCC specimens (arrows in Figure 1B–1D) inspired us to investigate the subcellular localization of p22phox when overexpressed in OSCC cells. By fluorescence microscopy, we found that the ectopically expressed p22phox (p22phox-DsRed) was mostly localized in the cytoplasm, forming a ring-like pattern at the nuclear periphery similar to that in CDDP-resistant OSCC tissues. To rule out the possibility of protein mislocalization due to the fluorescence tag, we asked whether the localization pattern could be reproduced in the parental OSCC cells transiently expressing non-tagged p22phox protein. Indeed, immunofluorescence microscopy showed a similar ring-like staining pattern around the nuclear membrane, albeit along with a rather diffuse cytoplasmic localization. Furthermore, such staining pattern was recapitulated in OSCC cells ectopically expressing HA-tagged p22phox (HA-p22phox) protein (Supplementary Figure 4). Since endogenous p22phox was present in a much lower level than the overexpressed p22phox and exhibited a diffuse localization pattern throughout the cell (Supplementary Figure 5), we speculated that the more abundantly p22phox was expressed, the more dramatically the ring-like pattern would form in both clinical tissues and cell lines of OSCC. However, the mechanism behind the transition from diffuse to intense perinuclear expression pattern is at present not understood. To further examine whether protein localization of p22phox would affect CDDP uptake and trafficking, we used fluorescence-labeled (Alexa Fluor 488) CDDP to monitor CDDP distribution in p22phox stable lines and KB carcinoma cell line transiently overexpressing p22phox [33]. Remarkably, we found that CDDP was co-localized with p22phox at the perinuclear and other cytoplasmic regions in both cell lines. Quantitative analysis revealed that, when compared to the control cells, the average cytoplasmic-to-nuclear ratio of Alexa Fluor 488-CDDP intensity was markedly higher in p22phox-overexpressing cells. It is thus conjectured that CDDP was sequestered by the overexpressed p22phox and accumulated in the cytoplasm, preventing the entry of CDDP into the nucleus. Interestingly, previous studies showed that cytoplasmic cysteine-rich proteins such as glutathione (GSH) and metallothioneins could robustly bind to and sequester CDDP, representing one of the known mechanisms of resistance to CDDP [34–36]. However, it is unlikely that p22phox and the cysteine-rich proteins share the same mechanism of CDDP resistance because of the distinct amino acid composition and cellular function; p22phox is no cysteine-rich protein and lack of known detoxification activity. Nonetheless, there was no direct evidence to prove how p22phox sequestered CDDP in the cytoplasm. Whether CDDP was constrained to the cytoplasm as a result of physical binding to the overexpressed p22phox has to be investigated. In addition, we studied whether the sequestration of CDDP in the cytoplasm would affect CDDP-DNA adduct formation in the nucleus. Indeed, CDDP-DNA adducts detected by dot blot analysis was significantly decreased in p22phox stable lines. Since chk1-p53 signaling has been shown to be activated during apoptosis triggered by CDDP-DNA adducts [37–39], we examined whether the reduced adduct formation would affect the response to DNA damage. Activation of the chk1-p53 DNA damage response signaling was attenuated throughout the CPPD treatment period in the stable lines. Together, these results indicated that overexpression of p22phox could cause defective entry of CDDP into the nucleus, significantly inhibiting adduct-induced apoptosis and contributing to drug resistance in OSCC cells.

**Figure 8:**
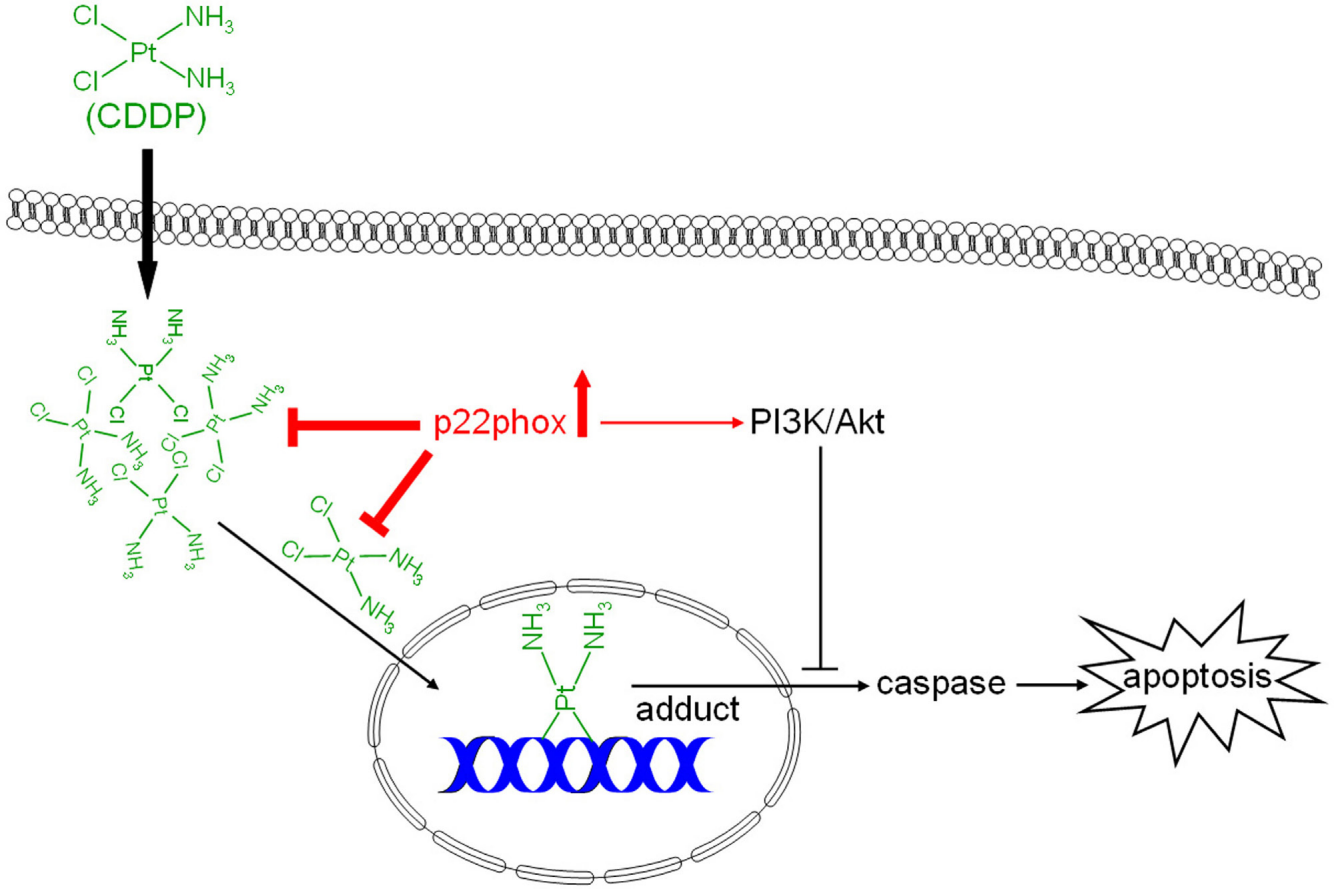
Dual modes of action of p22phox in the mechanism of CDDP resistance In OSCC cells, despite regular CDDP uptake into the cells, overexpression of p22phox possibly sequesters CDDP in the cytoplasm, resulting in diminished CDDP entry to and DNA adducts formation in the nucleus. The decreased DNA damage triggers attenuated apoptotic signaling that is further inhibited by p22phox-activating PI3K/Akt signaling pathway.

In conclusion, this study demonstrates for the first time that p22phox, a crucial component for NADPH oxidase activation, modulates CDDP resistance in OSCC cells. We found that p22phox was highly expressed in CDDP-resistant carcinoma tissues in oral cancer patients. *In vitro* studies demonstrated that overexpression of p22phox abrogated CDDP-induced apoptosis, thereby conferring cytoprotective effect against CDDP in OSCC cells. There were increased levels of basal Akt activity in p22phox stable lines, which only partially accounted for p22phox-dependent inhibition of CDDP-induced apoptosis. Furthermore, fluorescence-labeled CDDP was accumulated and co-localized with the overexpressed p22phox in the cytoplasm, which was supported by the reduced CDDP-adduct formation and chk1-p53 activation. Collectively, despite normal CDDP uptake into the cells, the entry of CDDP into the nucleus was severely impaired presumably due to sequestration by p22phox in the cytoplasm, eliciting a series of downstream effects including decreased DNA adduct formation and weakened apoptotic signaling. Finally the attenuated apoptosis was further inhibited by p22phox-activating PI3K/Akt pathway, thereby leading to CDDP resistance in OSCC cells (Figure 8). These data have provided a novel biomarker and insight into the mechanism of chemoresistance in oral cancer.

## MATERIALS AND METHODS

### Patients and tissue sample collection

Three paired CDDP-resistant and CDDP-sensitive formalin-fixed paraffin-embedded (FFPE) primary OSCC specimens were withdrawn from Chi-Mei Hospital Tissue BioBank approved by Institutional Review Board (IRB) of Chi-Mei Medical Center. Patients who received CDDP treatment alone (C/T) or together with radiotherapy (CCRT) and showed no recurrence for at least two years were defined as CDDP-sensitive, and were otherwise CDDP-resistant. Healthy oral mucosa was obtained during dental surgery. The clinicopathological data for the patients and samples were presented in Table 1.

### Immunohistochemistry

After the dewaxing and antigen retrieval, FFPE slides were serum-blocked (with normal rabbit serum) and incubated with anti-p22phox rabbit polyclonal antibody (Santa Cruz Biotechnology, Santa Cruz, CA) at 1:100 dilution for overnight at 4°C. The staining using streptavidin–biotin complex system and the scoring of immunoreactivity were the same as our previous publication [40].

### Cell lines and cell culture

Oral cancer cell lines SAS, Hep2, Ca9–22, HSC-3, CAL-27 and FaDu were obtained and maintained as mentioned previously [40]. Primary human oral keratinocytes (HOK) were purchased from ScienCell^TM^ Research Laboratories (Carlsbad, CA, USA) and maintained in Oral Keratinocyte Medium (OKM, Sciencell) with bovine pituitary extract (BPE)-containing growth supplements. HOK at the third passage were used in Western blot analysis.

### Antibodies, inhibitors and western blot analysis

Rabbit polyclonal anti-p22phox and anti-pAkt antibodies, and mouse monoclonal anti-p53 and anti-GAPDH antibodies were purchased from Santa Cruz Biotechnology (Santa Cruz, CA). Rabbit polyclonal antibodies against Akt, caspase 3, chk1 and p-chk1 were purchased from GeneTex (Irvine, CA), caspase 7, caspase 9, PARP and p-p53 were from Cell Signaling Technology (Danvers, MA), and DsRed was from BioVision (Mountain View, CA). PI3K inhibitors 3-methyladenine (3-MA) and wortmannin were purchased from Sigma, and Akt inhibitor (2E, 6E)-2,6-Bis(4-pyridinylmethylene) cyclohexanone (SC66) was from Tocris Bioscience (Bristol, UK). Western blot analysis was performed as previously described [40]. Quantification of the data was performed by densitometry.

### Transient siRNA knockdown

siGENOME SMARTpool siRNA against p22phox and Akt (siRNA of Akt1, Akt2 and Akt3) was purchased from Dharmacon (Lafayette, CO). Transfection of siRNA, 50 and 100 nM for p22phox and Akt, respectively, was performed according to our previous protocols [41]. Knockdown of p22phox and Akt expression was verified by Western blot analysis.

### Plasmid construction

p22phox-DsRed expression construct was obtained by PCR amplification of the p22phox coding sequence (CDS) from SAS cDNA pool using primer sequences: forward, 5′-CAGATCTCGAGATGGGGCAGATCGAGTGGG-3′ and reserve, 5′-CGGTGGATCCCGCACGACCTCGTC GGT-3′. The PCR fragments were digested with XhoI and BamHI, inserted into XhoI/BamHI site of pDsRed-N1 vector (Clontech Laboratories, Palo Alto, CA) and then sequenced. For cloning of p22phox into a non-tagged expression vector, p22phox CDS was PCR amplified using primer sequences: forward, 5′-CAGATGAATTCATGGGGCAGATCGAGTGGG-3′ and reverse, 5′-CGGTCTCGAGTCACACGACCTCGT CGGT-3′. The PCR products were cloned into EcoRI/XhoI site of pcDNA3.0 vector, generating pcDNA3.0-p22phox construct.

### Generation of p22phox stable lines

SAS cells were seeded into 10-cm culture dishes at a density of 1 × 10^6^ cells per dish. After attachment, the cells were transfected with 20 μg p22phox-DsRed construct or empty vector using electroporation (ECM830, BTX). Two days after transfection, the cells were re-plated in a 10-cm culture dish with medium containing 2 mg/ml G418. Two weeks later, the cells were sorted by FACS before isolation of single colonies. The isolated colonies were seeded initially into each well of the 24-well plates, followed by progressive expansion of the cultures in large culture dishes. G418-resistant stable clones were established and maintained in medium with 0.5 mg/ml G418. Stable clones with high p22phox expression (p22phox line #1 and #2) were verified by immunoblotting.

### MTT assay

Cells were seeded into 24-well plates at a density of 7 × 10^4^ in each well. After attachment, the cells were treated with various concentrations of CDDP for 48 h. Cell survival was evaluated by MTT assay (Chemicon International Inc., CA) based on previous protocol [40]. The values of IC_50_ were determined using CalcuSyn 2.1 software (Biosoft, Cambridge, UK).

### Assessment of cell cycle distribution

In brief, 7 × 10^5^ cells were treated with 10 μM CDDP for 24 h. These cells were then trypsinized, washed twice with PBS and fixed in 70% ethanol overnight. After centrifugation, the cell pellets were stained with 2 μg/ml 4′,6-diamidino-2-phenylindole (DAPI) (Roche Applied Science) for 30 min at 37°C in the dark. The cells were analyzed on a BD LSRII flow cytometer (BD Biosciences) and the results were evaluated using FCS Express 4 (De Novo Software, LA, CA).

### TUNEL assay

Detection of apoptotic cells was performed using APO-BrdU^TM^ TUNEL assay kit (Invitrogen). Briefly, 3 × 10^4^ cells were plated in each well of an 8-well Millicell EZ glass slide (Millipore, Billerica, MA). After treatment with CDDP (10 μM) for 48 h, the cells were fixed in 4% (w/v) paraformaldehyde on ice for 15 min and then washed twice with PBS. The fixed cells were stored in 70% ethanol at 4°C overnight followed by nicked ends labeling reaction according to the manufacturer's instructions. The positively labeled cells (green fluorescence) were visualized by fluorescence microscopy.

### Immunofluorescence

SAS cells (4 × 10^5^) were transfected with the expression constructs by liposome-based method (Lipofectamine 2000 Reagent, Invitrogen). One day later, the cells were trypsinized and allowed to attach onto coverslips for another 24 h. The cells were fixed with 4% paraformaldehyde for 15 min and then permeabilized with 0.1% Triton X-100 for another 15 min at room temperature. After washing three times with PBS, the cells were blocked with 2% BSA for 1 h. Primary antibody incubation (1:50 to 1:100 dilution) was done at 4°C overnight, followed by secondary antibody incubation with FITC-conjugated anti-mouse IgG or Texas Red-conjugated anti-rabbit IgG (1:200 dilution, Jackson Laboratories) for 50 min and DAPI stain (1 μg/ml) for 10 min at room temperature. Cells were mounted with anti-fading medium (Dako Fluorescence Mounting Medium) and visualized under a confocal microscope.

### Fluorescence detection of CDDP cell distribution

KB cells were transfected with p22phox-DsRed construct by electroporation. Twenty-four hours after transfection, the cells were trypsinized and seeded onto 8-well Millicell EZ glass slides and allowed to attach for another 24 h. The cells were then incubated with Alexa Fluor 488–CDDP (Molecular Probes, Eugene, OR) for 2 h, followed by a brief wash with PBS and fixation with 4% paraformaldehyde solution for 15 min at room temperature. The fixed cells were permeabilized with 0.1% Triton X-100 for 15 min and then stained with DAPI for 10 min. Detection of CDDP in p22phox stable line was identical to that in KB cells, except for the omission of the transfection step. The slides were mounted by anti-fading medium and observed using confocal microscopy. Quantitation of the fluorescence intensity was performed by Olympus Fluoview Viewer (Ver. 3.0 software).

### CDDP-DNA adduct detection

For genomic DNA isolation of CDDP- or mock-treated cells, 1 × 10^6^ of the cells were resuspended in 3 ml of TE buffer with 0.65% SDS. The cells were then digested with proteinase K (100 μg/ml) (Sigma) at 55°C overnight. Genomic DNA was precipitated by adding 1 ml of saturated NaCl and 10 ml of 100% ethanol (2.5 × volumes) followed by incubation at room temperature overnight. The DNA (5 μg) was spotted onto a nitrocellular membrane and incubated at 80°C for 2 h in an oven. The positive control was made by incubating the genomic DNA (5 μg) of control line with 100 μMCDDP at room temperature for 30 min. The blot was subjected to standard Western blot analysis using anti-CDDP adducts antibody (1:1000 dilution) (clone ICR4, Millipore, Temecula, CA) as mentioned.

### Statistical analysis

The cell survival data were evaluated by one-way ANOVA followed by LSD post-hoc comparison of p22phox siRNA vs. p22phox siRNA + CDDP and control siRNA + CDDP vs. p22phox siRNA + CDDP. The results of cytoplasm-to-nucleus fluorescence intensity ratio were further analyzed by Mann-Whitney *U* test. *P < 0.05 was considered statistically significant in all statistical assays.

## SUPPLEMENTARY FIGURES


