# Supplementary material for: p22phox confers resistance to cisplatin, by blocking its entry into the nucleus
Source: Oncotarget. 2015 Feb 19;6(6):4110–25. doi: 10.18632/oncotarget.2893 (PMC4414176; doi:10.18632/oncotarget.2893)
Supplement: Supplementary file 1 [file oncotarget-06-4110-s001.pdf]

SUPPLEMENTARY FIGURES

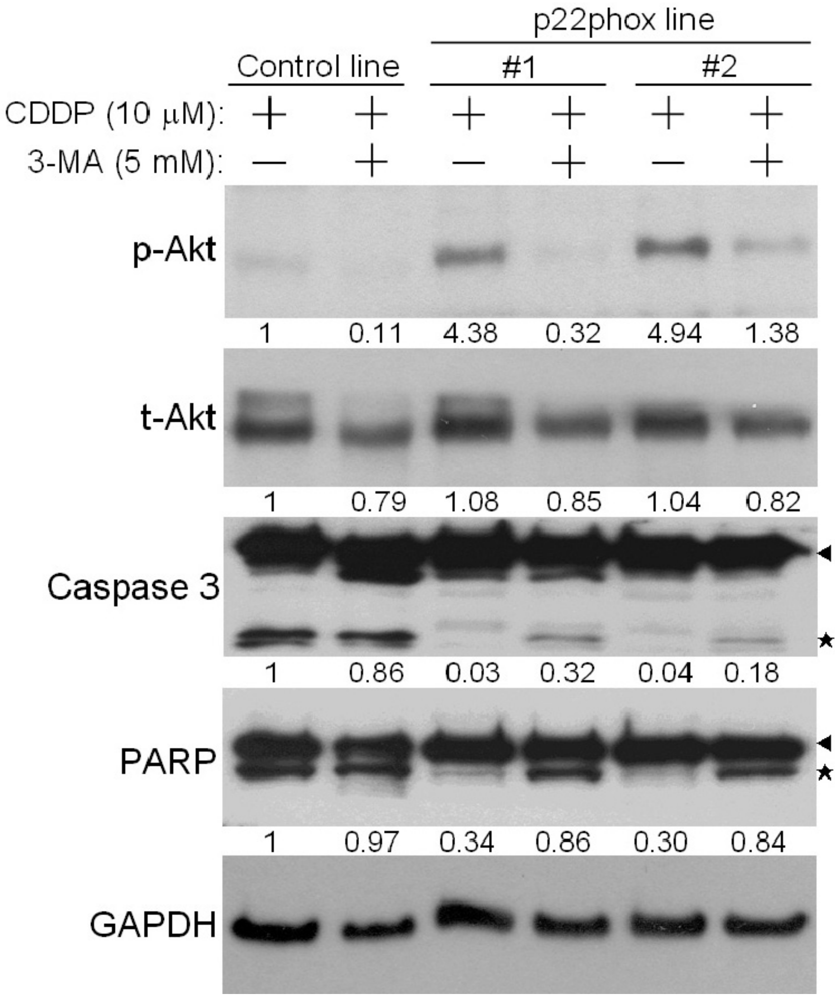

Supplementary Figure S1: CDDP-induced apoptotic signal was restored when PI3K/Akt pathway was blocked by PI3K inhibitor 3-MA in p22phox stable lines. Cells were pretreated with 3-MA (5 mM) overnight, followed by the combined treatment of CDDP (10 μM) and the inhibitor for another 24 h. Arrow heads and stars represent the pro-forms and the cleaved forms of caspase 3 and PARP, respectively. The numbers below the blots were quantitative ratios of p-Akt, t-Akt, cleaved caspase 3 or cleaved PARP/ GAPDH band intensities normalized to those without the inhibitor treatments in control line.

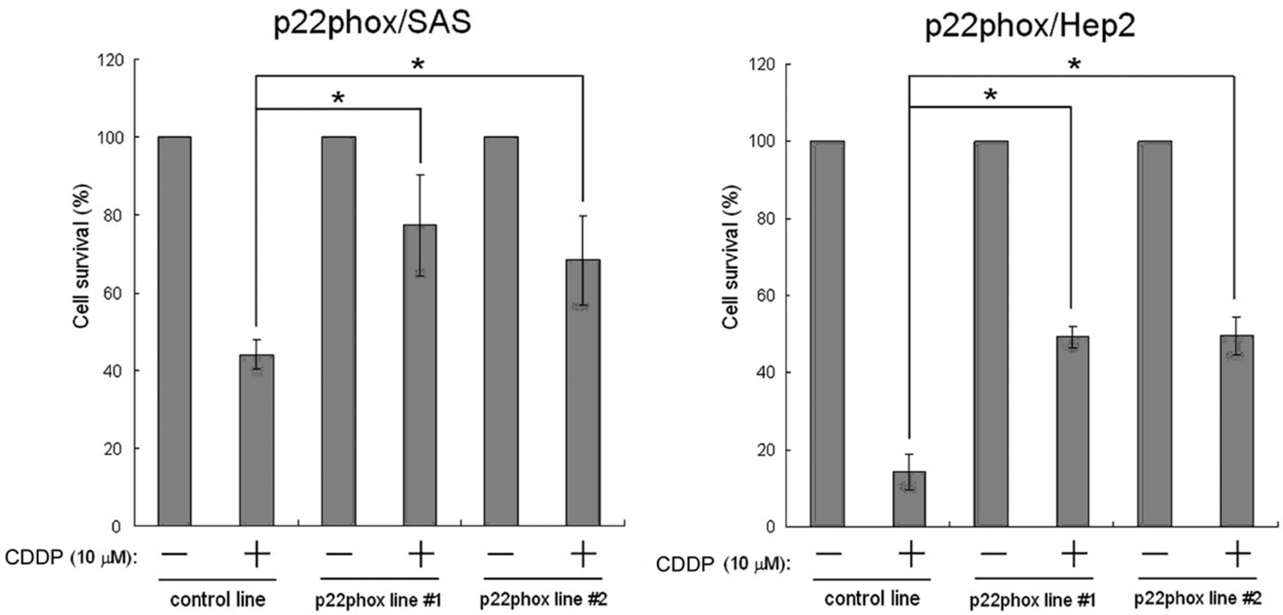

Supplementary Figure S2: Overexpression of p22phox in SAS and Hep2 increased cell survival under CDDP treatment. Oral cancer cell lines stably expressing p22phox (p22phox/SAS and p22phox/Hep2) were treated with the indicated concentrations of CDDP for 24 h. Survived cells were calculated by trypan blue staining. Two independent clones obtained from the establishment of each stable line were tested in these experiments. The control line was transfected with an empty expression vector and its viability was deliberately set to 100%. All measurements were done in triplicate and expressed as mean ± SD. The experiments were repeated at least three times and the representative data are shown. *$P < 0.01$ indicated significant increase compared to the control lines treated with CDDP.

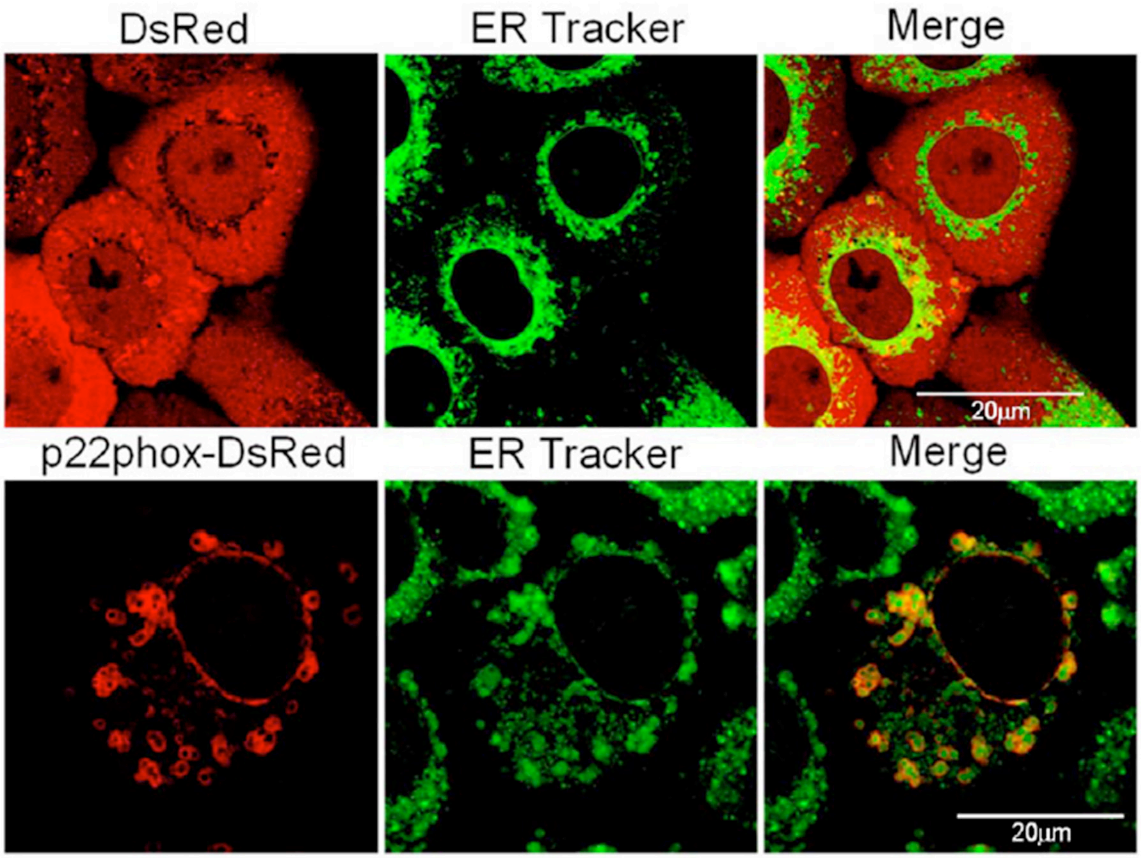

Supplementary Figure S3: Ectopically expressed p22phox (p22phox-DsRed) was co-localized with the endoplasmic reticulum (ER). The expression sites of DsRed and p22phox-DsRed proteins in the control line (upper panels) and p22phox stable line (lower panels), respectively, were revealed by fluorescence confocal microscopy. ER was stained by ER-Tracker™ (Invitrogen) (100 nM) for 30 min at room temperature. Magnification 1000X.

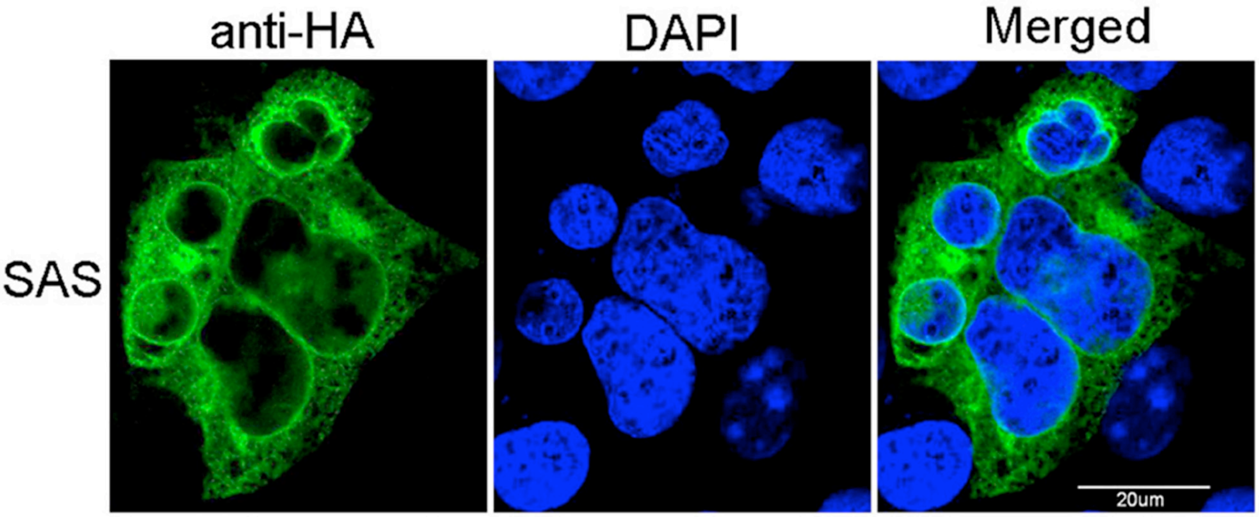

Supplementary Figure S4: Cellular localization of ectopically expressed HA-p22phox protein in OSCC cells. The overexpressed HA-p22phox protein in SAS cells was visualized by immunofluorescence and confocal microscopy using anti-HA antibody and FITC-conjugated anti-mouse IgG. There was diffuse staining mostly localized in the cytoplasm, with the unique ring-like pattern at the nuclear periphery. Magnification: 1000X.

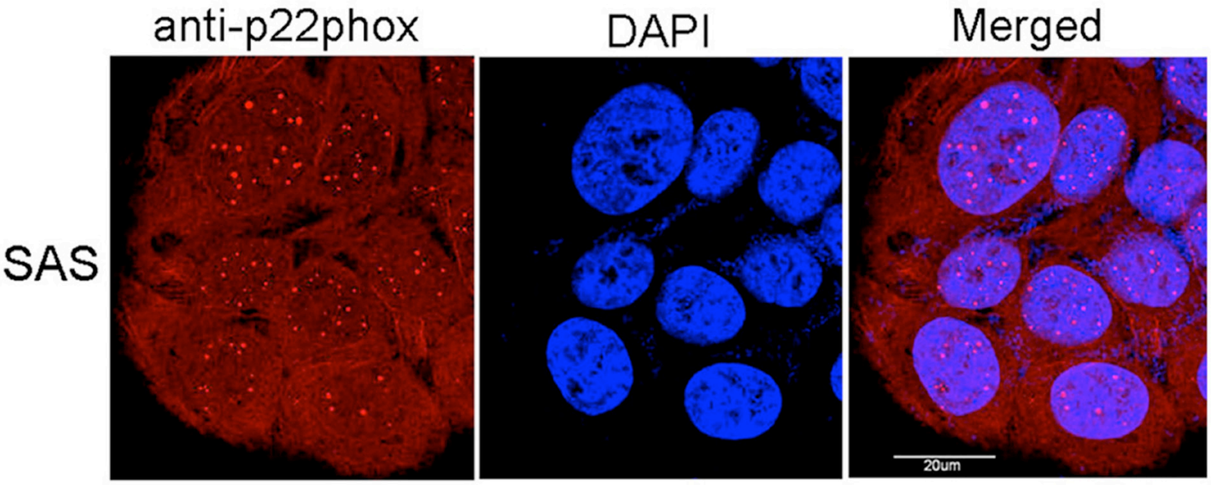

Supplementary Figure S5: Cellular localization of endogenous p22phox protein in OSCC cells. The endogenous p22phox expression in SAS cells was detected by immunofluorescence and confocal microscopy using anti-p22phox antibody and Texas Red-conjugated anti-rabbit IgG. There was diffuse staining throughout the entire cell, in which a unique speckled pattern was observed predominantly in the nucleus. Magnification: l000X.
